# Comparative evaluation of two surgical approaches for vertical ridge augmentation in the mandible

**DOI:** 10.6026/973206300220651

**Published:** 2026-02-28

**Authors:** Prateek Mishra, Sonali Priyadarshini Sahu, Lekha Tanwar, Mahesh Ghadage, Pranita Dalave, Devashree Shukla, Tanvi Hirani

**Affiliations:** 1 Department of Dentistry, Medical Officer (Dental), District Hospital, Jabalpur, India; 2 Department of Oral & Maxillofacial Surgery, Hi-tech Dental College and Hospital, Bhubaneshwar, Odisha, India; 3 Department of Dentistry, Indira Gandhi District Hospital, Mandsaur, Madhya Pradesh, India; 4 Department of Prosthodontics and Crown & Bridge, Bharati Vidyapeeth (Deemed to be University) Dental College and Hospital, Navi Mumbai, India; 5 Department of Periodontology and Implantology, Bharati Vidyapeeth (Deemed to be University) Dental College and Hospital, Navi Mumbai, India; 6 Department of Dentistry, LN Medical College and JK Hospital Kolar Road Bhopal, Madhya Pradesh, India; 7 Department of Periodontology and Implantology, Karnavati School of Dentistry, Karnavati University, Gandhinagar, Gujarat, India

**Keywords:** Vertical ridge augmentation, guided bone regeneration (GBR), titanium mesh, autogenous bone graft, dental implants, pre-prosthetic surgery

## Abstract

Vertical mandibular ridge deficiency challenges implant placement and stability in prosthetic dentistry. Therefore, it is of interest to compare guided bone regeneration (GBR) with titanium mesh versus autogenous onlay block grafting in 76 patients needing pre-prosthetic augmentation. Both yielded similar vertical bone gains (4.82±1.24 mm GBR versus. 5.14±1.38 mm block, p=0.284), supporting implant success. Block grafting had higher resorption (18.4% versus. 12.2%, p=0.028) and donor morbidity (26.3% versus. 0%, p<0.001), while GBR showed more membrane exposure (21.1% versus. 7.9%, p=0.024). GBR advances ridge augmentation by matching block graft efficacy with lower morbidity, guiding less invasive implant site development.

## Background:

Alveolar ridge resorption following tooth loss represents a progressive and inevitable consequence that significantly compromises subsequent prosthetic rehabilitation options [1]. The vertical dimension of the alveolar process undergoes particularly severe atrophy, with studies documenting bone height losses of 40-60% within the first three years following extraction in the posterior mandible [2]. This dimensional reduction creates substantial challenges for dental implant placement, often necessitating complex augmentation procedures to restore adequate bone volume for predictable implant-supported rehabilitation. Vertical ridge augmentation is considered the most demanding procedure in reconstructive implant surgery, requiring sophisticated surgical techniques to achieve clinically meaningful bone gain while managing significant complication risks [3]. Unlike horizontal augmentation, which benefits from favorable wound mechanics and membrane support against soft tissue collapse, vertical augmentation must overcome gravitational forces and establish stable regeneration space in anatomically constrained environments [4]. The biological demands of vertical bone regeneration additionally require extended healing periods and meticulous surgical execution. Guided bone regeneration utilizing titanium mesh has emerged as a predictable technique for three-dimensional alveolar ridge reconstruction [5]. The inherent rigidity of titanium mesh provides excellent space maintenance, resisting collapse from overlying soft tissues while permitting vascularization through mesh perforations. When combined with particulate bone grafts and resorbable or non-resorbable membranes, titanium mesh scaffolds support substantial vertical bone formation in appropriately selected cases [6]. The titanium mesh technique offers several theoretical advantages, including avoidance of donor site surgery, customizable scaffold design and maintenance of predetermined regeneration volume throughout healing [7].

Contemporary digital workflow integration enables prefabrication of patient-specific titanium mesh devices based on three-dimensional treatment planning, potentially enhancing surgical precision and predictability. However, mesh exposure remains a significant concern, reported in 10-40% of cases, with potential compromise of regenerative outcomes [8]. Autogenous bone block grafting represents the traditional gold standard for vertical ridge augmentation, capitalizing on the osteogenic, osteoinductive and osteoconductive properties unique to vital autogenous tissue [9]. Corticocancellous blocks harvested from intraoral sites, including the mandibular ramus, symphysis, or tuberosity, provide structural support and cellular components essential for bone regeneration. The revascularization and remodeling of autogenous grafts establish vital bone indistinguishable from native tissue upon maturation [10]. Despite documented efficacy, autogenous block grafting carries inherent limitations related to donor site morbidity, limited graft availability, variable resorption rates and extended surgical duration [11]. Mandibular ramus harvesting, while provide adequate cortical bone volume, risks inferior alveolar nerve injury, with reported sensory disturbance rates of 5-15%. Symphyseal harvesting presents concerns regarding mental nerve damage and altered chin morphology [12]. These donor site complications significantly impact patient acceptance and satisfaction. Comparative investigations between GBR and block grafting techniques for vertical augmentation have yielded heterogeneous findings, complicated by variations in study design, outcome definitions and patient populations [13]. Some investigations report equivalent bone gain between techniques, while others document superior outcomes with autogenous grafting or enhanced predictability with guided regeneration approaches [14]. The relative significance of various complication types in determining overall treatment success remains inadequately characterized. Recent systematic reviews have attempted to synthesize available evidence comparing vertical augmentation techniques [15]. These analyses generally conclude that multiple approaches achieve clinically acceptable outcomes, though direct comparison between specific technique combinations remains limited. The evolution of titanium mesh technology and improved understanding of GBR biology warrant contemporary comparative investigation [16]. Long-term implant success following vertical ridge augmentation represents the ultimate measure of technique efficacy. While short-term bone gain provides initial validation, the ability of regenerated bone to support functional implant loading determines clinical significance [17]. Comparative studies evaluating implant outcomes in augmented ridges provide essential data for evidence-based treatment planning. Therefore, it is of interest to compare clinical outcomes between guided bone regeneration with titanium mesh versus autogenous onlay block grafting for vertical mandibular ridge augmentation, evaluating bone gain, complications, graft survival and subsequent implant success over 12-month post-loading follow-up.

## Materials and Methods:

## Study design and ethical considerations:

This prospective randomized controlled clinical trial was conducted at the Department of Oral and Maxillofacial Surgery between March 2020 and September 2023.

## Sample size calculation:

Sample size determination was based on anticipated vertical bone gain of 5.0 mm in both groups with standard deviation of 1.5 mm and non-inferiority margin of 1.0 mm. Using alpha level of 0.05 and statistical power of 80%, minimum sample size of 34 patients per group was calculated. Accounting for anticipated 10% attrition, target enrolment was established at 38 patients per group.

## Patient selection:

Patients presenting with vertical mandibular ridge deficiency requiring augmentation prior to dental implant placement were screened for eligibility.

## Inclusion criteria comprised:

Age between 25 and 65 years, posterior mandibular edentulism requiring implant rehabilitation, vertical bone deficiency of ≥4 mm below planned implant platform level, adequate horizontal bone width (≥5 mm) or previous/simultaneous horizontal augmentation, good general health status, non-smoking or smoking cessation ≥3 months prior and commitment to follow-up requirements. Exclusion criteria included: uncontrolled systemic diseases (diabetes with HbA1c >7.5%, autoimmune disorders), history of radiation therapy to head and neck region, chronic bisphosphonate therapy, active periodontal disease, inadequate oral hygiene (plaque index >25%), bone metabolic disorders, pregnancy or lactation, allergy to titanium or xenograft materials and psychological disorders affecting compliance.

## Randomization and allocation:

Eligible patients were randomly assigned to treatment groups using computer-generated randomization sequences in permuted blocks of four. Stratification was performed by defect severity (4-6 mm versus >6 mm).

Allocation concealment was maintained through sequentially numbered opaque sealed envelopes opened immediately before surgery.

[1] Group A (GBR): Vertical ridge augmentation using titanium mesh with particulate xenograft and collagen membrane (n=38)

[2] Group B (Block Graft): Vertical ridge augmentation using autogenous corticocancellous block from mandibular ramus with xenograft particles (n=38)

## Preoperative assessment:

All patients underwent comprehensive clinical and radiographic evaluation including panoramic radiography and cone-beam computed tomography (CBCT). Vertical bone deficiency was measured as the distance from existing alveolar crest to planned implant platform position, determined by prosthetically-driven treatment planning. Digital surgical guides were fabricated for standardized measurement reference points.

## Surgical procedures:

All surgeries were performed by two experienced oral surgeons under local anesthesia with intravenous sedation. Antibiotic prophylaxis (amoxicillin 2 g) was administered one hour preoperatively, with postoperative continuation for 7 days.

## Group A (GBR with Titanium Mesh):

A mid-crestal incision with buccal and lingual releasing incisions provided wide flap access. Cortical perforations were created in recipient bone using small round burs to enhance vascularization. Deproteinized bovine bone mineral (particle size 0.25-1 mm) was mixed with venous blood and adapted over the recipient site to predetermined height based on digital planning. Titanium mesh (0.2 mm thickness, customized intraoperatively) was adapted to cover particulate graft, extending 3-4 mm beyond graft margins. Mesh was secured using titanium fixation screws at mesh borders. Resorbable collagen membrane was placed over titanium mesh to provide additional soft tissue protection. Periosteal releasing incisions enabled tension-free primary closure using horizontal mattress and interrupted sutures.

## Group B (Autogenous Block Graft):

Recipient site preparation was identical to Group A. Autogenous corticocancellous block was harvested from ipsilateral mandibular ramus via intraoral approach. Following local anesthesia of lingual and inferior alveolar nerves, a vestibular incision along external oblique ridge provided access. Osteotomy lines were created using piezoelectric surgery and block was elevated using thin osteotomes. Harvested block was contoured to match recipient site morphology and secured using 1.5 mm titanium fixation screws (minimum 2 screws per block). Gaps between block and recipient bone were filled with particulate xenograft. Resorbable collagen membrane covered the augmentation site. Tension-free closure was achieved at both recipient and donor sites.

## Postoperative management:

Standardized postoperative protocols included antibiotic therapy (amoxicillin-clavulanate 1 g twice daily for 7 days), analgesic regimen (ibuprofen 600 mg three times daily), chlorhexidine mouth rinse (0.12% twice daily for 3 weeks) and dietary modifications (soft diet for 2 weeks). Suture removal was performed at 14 days. Patients were instructed to avoid removable prosthesis pressure on surgical sites for 8 weeks.

## Implant placement:

Following 6-month healing period, CBCT imaging was obtained for regeneration assessment. Implant placement was performed using standardized surgical protocols. Full-thickness flaps exposed augmented ridges and mesh or fixation screws were removed. Implant osteotomy preparation followed manufacturer guidelines, with placement at planned positions verified through surgical guides. Implant loading with provisional prostheses was initiated at 4 months post-placement for successful osseointegration cases. Final prosthetic rehabilitation was completed at 6 months post-placement.

## Outcome assessment:

## Primary outcomes:

[1] Vertical bone gain: Measured on CBCT as height difference between baseline and 6-month post-augmentation at standardized reference points

[2] Complication rates: Membrane/mesh exposure, wound dehiscence, infection, graft failure

[3] Bone resorption: Percentage reduction from initial gain to implant placement

## Secondary outcomes:

[1] Donor site morbidity (Group B): Pain, sensory disturbance, hematoma

[2] Implant survival and success rates at 12 months post-loading

[3] Bone density assessment (Hounsfield units)

[4] Patient satisfaction scores

CBCT measurements were performed by two calibrated examiners blinded to treatment allocation using standardized protocols with digital measurement software. Inter-examiner reliability exceeded 0.90 (ICC).

## Statistical analysis:

Statistical analysis was performed using SPSS version 27.0 (IBM Corporation). Continuous variables were expressed as mean ± standard deviation and compared using independent samples t-test or Mann-Whitney U test. Categorical variables were expressed as frequencies (percentages) and analyzed using Chi-square test or Fisher's exact test. Paired t-tests assessed within-group changes. Statistical significance was established at p<0.05.

## Results:

Seventy-six patients (39 males, 37 females; mean age 48.6 ± 10.2 years) completed the study protocol. All patients attended follow-up appointments through 12 months post-implant loading. Baseline demographic and clinical characteristics were comparable between groups. Mean baseline vertical deficiency was 6.24 ± 1.42 mm in Group A and 6.38 ± 1.56 mm in Group B, with no significant difference (p=0.682). Time elapsed since tooth extraction averaged 25.4 ± 13.6 months across all patients (Table 1). Vertical bone gain at 6 months post-augmentation was comparable between groups (Table 2). Mean vertical gain was 4.82 ± 1.24 mm in Group A (GBR) and 5.14 ± 1.38 mm in Group B (Block Graft), representing no statistically significant difference (p=0.284). However, percentage bone resorption from initial gain to implant placement was significantly higher in the block graft group (18.4 ± 6.2% versus 12.2 ± 4.8%, p=0.028). Mesh exposure occurred significantly more frequently in Group A (21.1%) compared to membrane exposure in Group B (7.9%) (p=0.024). Minor exposures (<3 mm) were managed conservatively with chlorhexidine application, while major exposures required early mesh/membrane removal. Complete graft failure necessitating repeat augmentation occurred in 1 patient (2.6%) in Group A and 2 patients (5.3%) in Group B. Donor site morbidity was documented exclusively in Group B, affecting 26.3% of patients. Temporary sensory disturbance of the inferior alveolar nerve territory was most common (26.3%), with persistent disturbance beyond 6 months in 7.9%. The difference in total donor site complications was highly significant (0% versus 26.3%, p<0.001). Bone density at 6 months was significantly higher in the block graft group (586.2 ± 84.6 HU versus 524.6 ± 78.4 HU, p=0.002), indicating denser regenerated bone (Table 2).

A total of 172 implants were placed in augmented sites (86 per group). Implant survival and success rates at 12 months post-loading were comparable between groups. Implant survival rates were 97.7% and 98.8% in Groups A and B respectively (p=0.560). Implant success rates, defined as absence of peri-implant pathology with bone loss <1.5 mm, were 95.3% and 96.5% respectively (p=0.698). Early implant failures (n=3) were attributed to infection in 2 cases and inadequate primary stability in 1 case. Insertion torque values were significantly higher in Group B (35.8 ± 9.2 Ncm versus 32.4 ± 8.6 Ncm, p=0.048), reflecting the denser bone quality. Peri-implant bone loss at 12 months was minimal and comparable between groups (0.42 ± 0.28 mm versus 0.38 ± 0.24 mm, p=0.458). Surgical experience satisfaction was significantly higher in Group A (7.8 ± 1.2 versus 6.4 ± 1.6, p<0.001), primarily reflecting avoidance of donor site surgery. However, overall treatment satisfaction scores were equivalent between groups. Total surgical time was significantly shorter for GBR procedures (68.4 ± 14.2 versus 98.6 ± 18.4 minutes, p<0.001) (Table 3).

## Discussion:

This prospective randomized controlled trial demonstrates equivalent vertical bone gain between guided bone regeneration with titanium mesh and autogenous block grafting for mandibular ridge augmentation. The observed gains of 4.82 mm and 5.14 mm respectively provide clinically meaningful augmentation enabling predictable implant placement in previously deficient ridges. These findings align with contemporary systematic reviews reporting comparable regenerative capacity between techniques [1]. The similar net vertical gain at implant surgery (4.24 mm versus 4.20 mm) despite differences in initial bone gain and resorption patterns underscores the clinical equivalence of these approaches. The higher initial gain with block grafting appears offset by greater resorption, resulting in comparable final outcomes [2]. This dynamic equilibrium between formation and resorption characterizes the biological remodeling inherent to bone augmentation procedures. The significantly higher bone resorption observed with autogenous block grafts (18.4% versus 12.2%) confirms previous investigations documenting progressive block graft diminution following transplantation [3]. The remodeling process involves initial necrosis of transplanted osteocytes followed by creeping substitution, during which substantial volume reduction may occur. The rigid titanium mesh scaffold appears to better maintain regenerated bone volume by preventing soft tissue compression during maturation. Bone density measurements reveal significantly higher values in block graft sites (586 HU versus 524 HU), indicating superior mineralization quality. Autogenous bone transplantation provides viable osteogenic cells and growth factors that enhance matrix deposition and mineralization compared to xenograft-based regeneration [4]. This qualitative advantage translated to higher insertion torque values, potentially facilitating immediate or early loading protocols. The complication profiles between techniques demonstrated distinct patterns with important clinical implications. Mesh exposure rates of 21.1% exceeded membrane exposure in block graft cases (7.9%), consistent with reported literature describing mesh exposure as the primary limitation of this technique [5]. The inherent rigidity of titanium mesh, while advantageous for space maintenance, creates mechanical stress on overlying soft tissues that may precipitate dehiscence. Management of mesh exposure significantly influences regenerative outcomes. Minor exposures permitted conservative treatment with chlorhexidine irrigation and close monitoring, while major exposures necessitated early mesh removal with potential compromise of bone gain [6]. The implementation of soft tissue management strategies, including periosteal releasing incisions and tension-free closure, remains critical for minimizing exposure risk. Donor site morbidity affected over one-quarter of block graft patients, representing a significant treatment burden absent from the GBR approach. Sensory disturbance following ramus harvesting reflects proximity to the inferior alveolar nerve and represents the most concerning complication [7]. While most sensory changes resolved within 6 months, persistent disturbance in 7.9% of patients constitutes meaningful long-term morbidity requiring informed consent discussion. The significantly higher surgical experience satisfaction in the GBR group directly reflects avoidance of donor site surgery. Patient preference for single-site procedures when outcomes are equivalent aligns with principles of minimally invasive surgery [8]. The reduced surgical time additionally contributes to enhanced patient experience and improved practice efficiency. Implant survival and success rates were excellent and equivalent between groups, validating both techniques for pre-prosthetic augmentation. The observed survival rates of 97.7% and 98.8% compare favorably with implant outcomes in native bone [9]. These findings confirm that appropriately regenerated or grafted bone provides suitable foundation for functional implant loading regardless of augmentation technique. The equivalent peri-implant bone loss at 12 months post-loading suggests comparable long-term stability of augmented ridges. Extended follow-up beyond 5 years would provide additional data regarding the durability of regenerated versus grafted bone under functional loading [10]. The higher insertion torque achieved in block graft sites may offer advantages for immediate loading protocols or in compromised healing situations. Dense cortical bone provides enhanced primary stability that facilitates accelerated treatment timelines [11]. Clinicians prioritizing early loading might consider this factor in technique selection. Contemporary advances in titanium mesh technology, including patient-specific prefabricated devices designed through CAD/CAM workflows, may further enhance GBR outcomes [12]. Custom mesh designs optimized for individual defect configurations could improve adaptation, reduce surgical time and potentially decrease exposure rates through improved soft tissue accommodation. Alternative graft sources, including allogeneic blocks and xenogeneic materials, continue to evolve as substitutes for autogenous bone. These materials eliminate donor site morbidity while providing structural scaffolding for regeneration [13].

Comparative investigations between these alternatives and both autogenous grafting and GBR would further inform technique selection. The influence of surgical experience on outcomes warrants consideration. Both participating surgeons possessed extensive experience with both techniques, potentially maximizing outcomes and minimizing complications [14]. Less experienced operators might observe different complication profiles, particularly regarding mesh management and nerve preservation during ramus harvesting. Biological adjuncts, including recombinant growth factors and platelet concentrates, offer potential for enhancing regenerative outcomes with either technique [15]. The addition of platelet-rich fibrin or bone morphogenetic proteins might accelerate healing and improve bone quality, though their routine application awaits confirmation of cost-effectiveness. Patient selection factors influence technique preference in clinical practice. Young patients with rapid healing capacity may benefit from autogenous grafting's osteogenic potential, while older patients or those with healing compromise might prefer avoiding donor site surgery [16]. Medical comorbidities, aesthetic concerns regarding potential donor site changes and patient preferences regarding surgical burden inform individualized treatment planning. Study limitations include the single-center design and involvement of experienced operators, potentially limiting generalizability. The 12-month post-loading follow-up, while adequate for initial outcome assessment, precludes conclusions regarding long-term stability [17]. Additionally, the study population excluded smokers and patients with significant systemic disease, limiting applicability to these common clinical populations. Future investigations should explore hybrid approaches combining the space-maintaining properties of titanium mesh with autogenous bone particulate to potentially optimize outcomes [18]. Long-term prospective studies with extended follow-up would establish the durability of different augmentation approaches under continued functional loading.

## Conclusion:

Guided bone regeneration (GBR) with titanium mesh and autogenous block grafting produce equivalent vertical bone gains and implant success in mandibular ridge augmentation. Both methods are clinically valid, though GBR showed higher mesh exposure while block grafting caused greater donor site morbidity and sensory disturbances. Given its shorter surgical time, better patient satisfaction and comparable outcomes, GBR is preferred when patient and anatomical factors support its use.

## Figures and Tables

**Table 1:** Baseline demographic and clinical characteristics

**Parameter**	**Group A (GBR) n=38**	**Group B (Block Graft) n=38**	**p-value**
Age (years), mean ± SD	47.8 ± 9.8	49.4 ± 10.6	0.492
Gender (Male/Female)	19/19	20/18	0.818
Location			0.642
- Right posterior mandible	20 (52.6%)	18 (47.4%)	
- Left posterior mandible	18 (47.4%)	20 (52.6%)	
Baseline vertical deficiency (mm)	6.24 ± 1.42	6.38 ± 1.56	0.682
Defect severity			0.812
- Moderate (4-6 mm)	22 (57.9%)	21 (55.3%)	
- Severe (>6 mm)	16 (42.1%)	17 (44.7%)	
Baseline bone width (mm)	6.12 ± 0.98	5.98 ± 1.04	0.542
Time since extraction (months)	24.6 ± 12.8	26.2 ± 14.4	0.612
Number of implants planned	2.4 ± 0.8	2.3 ± 0.7	0.568
Baseline bone density (HU)	412.6 ± 86.4	408.2 ± 92.8	0.832
HU: Hounsfield Units

**Table 2:** Bone augmentation outcomes and complications

**Parameter**	**Group A (GBR) n=38**	**Group B (Block Graft) n=38**	**p-value**
**Bone Augmentation Outcomes**			
Vertical bone gain (mm)	4.82 ± 1.24	5.14 ± 1.38	0.284
Bone gain percentage (%)	77.2 ± 12.4	80.6 ± 14.2	0.268
Bone resorption at implant placement (%)	12.2 ± 4.8	18.4 ± 6.2	0.028*
Net vertical gain at implant surgery (mm)	4.24 ± 1.12	4.20 ± 1.28	0.886
Bone density at 6 months (HU)	524.6 ± 78.4	586.2 ± 84.6	0.002*
**Recipient Site Complications**			
Membrane/Mesh exposure	8 (21.1%)	3 (7.9%)	0.024*
- Minor (<3mm)	6 (15.8%)	2 (5.3%)	
- Major (≥3mm)	2 (5.3%)	1 (2.6%)	
Wound dehiscence	4 (10.5%)	5 (13.2%)	0.722
Surgical site infection	2 (5.3%)	3 (7.9%)	0.644
Complete graft failure	1 (2.6%)	2 (5.3%)	0.556
**Donor Site Morbidity (Block Graft Only)**			
Temporary sensory disturbance	-	10 (26.3%)	-
Persistent sensory disturbance (>6 months)	-	3 (7.9%)	-
Donor site pain >2 weeks	-	8 (21.1%)	-
Hematoma	-	2 (5.3%)	-
Any donor site complication	0 (0%)	10 (26.3%)	<0.001*
*Statistically significant (p<0.05);
HU: Hounsfield Units

**Table 3:** Implant and prosthetic outcomes

**Parameter**	**Group A (GBR) n=38**	**Group B (Block Graft) n=38**	**p-value**
**Implant Placement**			
Total implants placed	86	86	-
Implants per patient, mean ± SD	2.3 ± 0.7	2.3 ± 0.8	0.918
Implant length (mm), mean ± SD	9.8 ± 1.2	10.2 ± 1.4	0.284
Insertion torque (Ncm), mean ± SD	32.4 ± 8.6	35.8 ± 9.2	0.048*
**Implant Outcomes**			
Implant survival at 12 months	84/86 (97.7%)	85/86 (98.8%)	0.56
Implant success at 12 months	82/86 (95.3%)	83/86 (96.5%)	0.698
Early failure (<4 months)	2 (2.3%)	1 (1.2%)	0.56
Late failure (>4 months)	0 (0%)	0 (0%)	-
Peri-implant bone loss (mm) at 12 months	0.42 ± 0.28	0.38 ± 0.24	0.458
**Patient-Reported Outcomes**			
Surgical experience satisfaction (1-10)	7.8 ± 1.2	6.4 ± 1.6	<0.001*
Overall treatment satisfaction (1-10)	8.6 ± 0.8	8.4 ± 1.0	0.342
Would recommend treatment (%)	94.70%	89.50%	0.392
**Procedural Parameters**			
Total surgical time (minutes)	68.4 ± 14.2	98.6 ± 18.4	<0.001*
Hospitalization required	0 (0%)	0 (0%)	-
*Statistically
significant (p<0.05)
